# Target detection in healthy 4-week old piglets from a passive two-tone auditory oddball paradigm

**DOI:** 10.1186/s12868-020-00601-4

**Published:** 2020-12-07

**Authors:** R. Anna Oeur, Susan S. Margulies

**Affiliations:** 1 Wallace H. Coulter Department of Biomedical Engineering, Emory University, 615 Michael St. Suite 655, Atlanta, GA USA; 2 Emory University, Health Sciences Research Building 1760 Haygood Drive, Suite W242, 30322 Atlanta, Georgia

**Keywords:** Auditory oddball paradigm, EEG, Event-related potential, Porcine

## Abstract

**Background:**

Passive auditory oddball tests are effort independent assessments that evaluate auditory processing and are suitable for paediatric patient groups. Our goal was to develop a two-tone auditory oddball test protocol and use this clinical assessment in an immature large animal model. Event-related potentials captured middle latency P1, N1, and P2 responses in 4-week old (N = 16, female) piglets using a custom piglet 32- electrode array on 3 non-consecutive days. The effect of target tone frequency (250 Hz and 4000 Hz) on middle latency responses were tested in a subset of animals.

**Results:**

Results show that infrequent target tone pulses elicit greater N1 amplitudes than frequent standard tone pulses. There was no effect of day. Electrodes covering the front of the head tend to elicit greater waveform responses. P2 amplitudes increased for higher frequency target tones (4000 Hz) than the regular 1000 Hz target tones (p < 0.05).

**Conclusions:**

Two-tone auditory oddball tests produced consistent responses day-to-day. This clinical assessment was successful in the immature large animal model.

Highlights

- Two-tone auditory oddball tests were successfully assessed in an immature large animal model.
- Consistent N1, P2 amplitudes were observed across three non-consecutive test days.
- Infrequent target tone pulses elicited greater amplitude responses than frequent standard tone pulses.

## Background

Approximately 283,000 children and adolescents (< 18 years old) visit the emergency department for a sports related traumatic brain injury (TBI) [1]. Considering the number of people who fail to seek medical care due to the seemingly ‘mild’ nature of some brain injury symptoms, the incidence of sports related TBI is likely much higher and has been estimated to be between 1.3 and 3.8 million [2]. The primary cause of non-fatal TBI for those 0–4 years old are from falls, and those 5–19 years old are from sport and recreation activities [3]. Mild TBI diagnosis relies on self-reported signs and symptoms and voluntary participatory assessments [4] resulting in greater challenges capturing accurate reporting and evaluation in the young paediatric population. There is heightened concern for mTBI in this age group due to the potential long-term neurological sequelae affecting cognition and behavior hindering learning and development, thus supporting the need for objective biomarkers of mTBI that are not dependent upon patient reporting and effort [5].

Electroencephalography (EEG) is a promising tool in the study of neurological diseases as it measures the electrical potential of the brain on the millisecond scale and provides a real-time assessment of neural processes. Event related potentials (ERPs), measure brain-related activity in response to a stimulus and relates cerebral function with deficits and injury outcomes [6, 7]. Auditory ERPs from infants and children in response to speech and sound processing have been correlated with the development of language suggesting that early differentiation of speech sounds have favorable associations with learning and reading capability [8–10]. In addition, auditory ERPs have been used as indices marking severe language impairment in children ages 9–15 years old in comparison to age matched controls [11]. Auditory oddball paradigms and ERPs are common tests used to elicit auditory processing at the cortical level [12, 13], and have been used as a marker of altered cognition in various diseased populations including concussion [14], schizophrenia [15] and autism [16]. These tests are effort independent tests that present infrequent ‘target’ tone pulses amongst a series of more frequent ‘standard’ tone pulses. Typically, the target tone elicits greater electrical potentials reflected as larger magnitude responses in comparison to the standard tones. Common response characteristic of the auditory oddball paradigm reflected in the EEG waveform are a series of positive peaks (P) and negative troughs (N) subsequently labelled in ascending temporal order (P1, N1, P2) or based on time course (P50 at 50 ms). Auditory stimulation causes an early positive peak, P1 or P50 around 50 ms and is associated with an orientation to a new sound, not yet influenced by attention. What follows is a negative peak around 100 ms (N1), thought to be associated with early attention and related to detecting sensory changes. A second positive peak at 200 ms (P2) is also considered to be involved in early attention [12, 13].

Findings in the literature for concussed patient groups have reported attenuated amplitudes and longer latency responses in comparison to a healthy cohort [17]. The on-going hypothesis is that the altered brain is unable to mobilize attentional resources to elicit similar magnitude responses (reduced amplitudes) and longer latencies indicate slower processing speeds [12]. Research findings have not been congruent across studies where some have demonstrated no differences in ERPs between injured and healthy cohorts, which may be a result of subtleties in the auditory oddball paradigm employed [14]. Passively listening to tones versus more complex oddball tasks requiring active participation through counting or pressing a button at the presentation of a target stimulus can involve different auditory pathways [18]. Despite more complex tasks revealing subtleties in auditory deficits after mTBI [19, 20], these participatory assessments may present an added challenge in the paediatric patient.

In addition to non-uniform test protocols and stimulus paradigms employed across studies, diverse findings can also be attributed to participant ages, sample sizes and the mechanisms or causes of head trauma [21, 22]. The mechanism of injury plays a primary role in the patterns of trauma where biomechanical characteristics such as the direction of head movement and relative levels of head rotation influence the nature and distribution of neural tissue traumas and subsequent injury outcomes [23, 24]. Varying levels of these injury factors likely contribute to the specific brain structures and functions that result in diverse signs and symptoms associated with mTBI, possibly leading to sub-types of concussion [25].

Preclinical animal models allow control of a number of factors, including biomechanical loading characteristics that permit systematic evaluation of structural, functional, and behavioural outcomes. Pigs are a common large animal model used to study neurological disorders and TBI [26, 27]. The 4-week old piglet brain is an established model of paediatric TBI and has been previously used to study diffuse axonal injury and intracranial haemorrhage [28, 29]. The anatomy of the pig brain contains similar distributions of white and grey matter, in addition to well-formed sulci and gyri that are key to modelling the human brain [30, 31]. The maturation trajectory of piglet brains are also similar to the young human brain with the 3–4 week old and 3 month old piglet paralleling the child and adolescent brain [32–34].

The objective of this study is to establish the auditory oddball response, a common study paradigm used in humans, in the 4-week old piglet model to benchmark auditory processing in a healthy cohort. It is hypothesized that P1, N1, P2 features will be observable in the waveform responses, and that the infrequent randomized target tones produce a greater response than the frequent standard tones. A second objective was to examine the effect of target tone frequency. In a separate set of tests, the frequency of the target tone was four times lower (250 Hz) or higher (4000 Hz) than the regular target tone (1000 Hz). The standard tone in all cases was 800 Hz. It is hypothesized that the greater the 4000 Hz target tone would produce a greater P1, N1, and P2 amplitudes than the 250 and 1000 Hz target tones.

## Methods

All procedures were approved by the Institutional Animal Care and Use Committee (IACUC) at Emory University School of Medicine. Experiments were carried out in an AAALAC International (Association for Assessment and Accreditation of Laboratory Animal Care) accredited facility. Sixteen 4-week-old female Yorkshire piglets (*Sus Sus scrofa*) were acquired from a commercial vendor, Palmetto Research Swine (South Carolina, USA) and were studied in this research to understand auditory processing in a healthy cohort. The animals were housed in groups of two to four in metal enclosures with plastic slated floors to allow for socialization. All animals were on a 12-h light and 12-h dark cycle and permitted *ad libitum* access to mini pig start diet (LabDiet 5080, St. Louis, MO) and water. All data collection was performed in a separate test room that housed all necessary equipment. Prior to auditory tests, animals were acclimated to the test room, sling, and a nylon stocking to simulate the EEG net. Acclimation was repeated on two separate days. On test days, animals were taken one at a time into the test room, placed in a sling, and were closely monitored and supported by two research staff.

EEG were collected using a custom-built 32-electrode Hydrocel Geodesic Sensor Net (GSN), originally designed for use on humans, that recorded scalp electrical activity during auditory stimuli (Electric Geodesics Inc., EGI, Eugene, OR). The electrode net is an elastomer structure with embedded chambers containing a single electrode and sponge, with room for eyes and ears and adjustable chinstraps to ensure a close fit. A computer cart with a Hypertronics connector arm links the 32-electrode net to the Net Amps 400 amplifier (Electric Geodesics Inc., EGI, Eugene, OR) and houses the data acquisition and stimulus presentation computers. Data acquisition was accomplished using a MacBook Pro laptop with Netstation 5.0 software (Electric Geodesics Inc., EGI, Eugene, OR) synchronized to a PC computer with E’ Prime 2.0 (Psychology Software Tools, Inc, Pittsburgh, PA) stimulus presentation software. A portable speaker connected to the PC computer via audio jack played the auditory stimuli at a distance of approximately 0.5 m from the top and centre of the piglets head. Prior to testing, the electrode net was soaked in a solution of baby shampoo (5 mL) and potassium chloride (10 mL) mixed in 1 L of water for at least 5 min. The net was then applied to the surface of the animal’s head and electrical impedance for all electrodes was checked to be below 1 kΩ. A light nylon stocking with holes cut out for ears and eyes was fitted over the electrode net to maintain scalp contact throughout data collection.

### Part A—regular oddball clicktrain

**Fig. 1:**
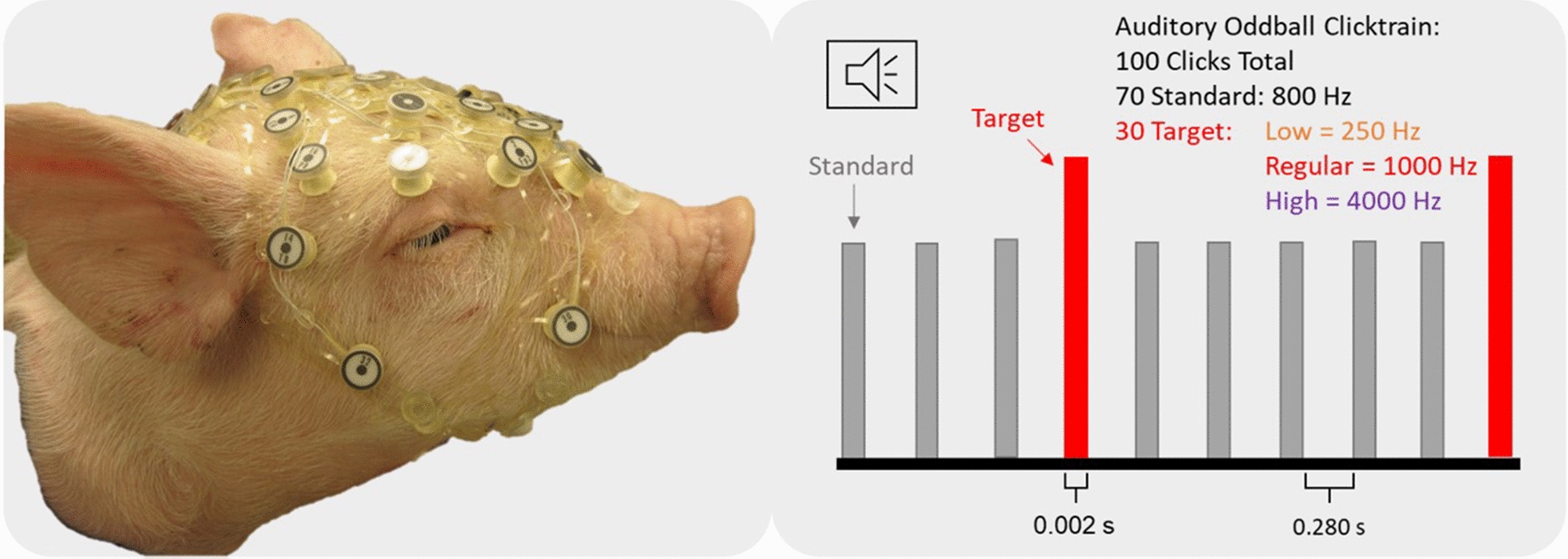
Depiction of an animal fitted with the 32-electrode net (left) and a representation of the auditory oddball stimulus presentation sequence (right)

EEG data was collected for each animal in response to an auditory oddball paradigm comprised of 100 tone pulses with each pulse lasting 2 ms and an inter-stimulus interval of 280 ms. The ‘regular’ oddball clicktrain was comprised of 70 standard 800 Hz tone pulses and 30 target 1000 Hz tone pulses played in random order (Fig. 1). Each 100-pulse clicktrain lasted approximately 30 s and was repeated for each animal to obtain 6 good trials. Twelve piglets (N = 12) were tested according to the ‘regular’ oddball click train for three non-consecutive days of testing.

### Part B—alternative target pulse clicktrain

A subset of six animals (out of twelve from *Part A)* were played an alternative clicktrain on a fourth day where the pitch of the target was either a low 250 Hz target tone pulse (3 animals) or a high 4000 Hz target tone pulse (3 animals). Four additional animals were studied and subject to the regular oddball clicktrain, followed by a low target pulse click train (2 animals) or a high target pulse click train (2 animals). All of these tests occurred on the same day, where the regular oddball was played first, followed by the alterative oddball clicktrain to keep with a similar order as the subset of six animals studied from *Part A.* In total, five animals were tested according to regular and low target (N = 5) pulse clicktrains and five animals were tested with regular and high target (N = 5) pulse clicktrains. All animal data from both the regular and the alternative oddball click trains were processed and there were no exclusions from analysis. All animals were euthanized within 3 days after the last study day with an overdose of pentobarbital (Euthasol 1 mL/10 lb of body weight) via an intracardiac injection. Prior to the injection, all piglets were under general anaesthesia using isoflurane at a rate of 3–5% and the absence of a deep pain response was confirmed using a toe pinch.

### Data processing

Data were processed using Netstation Tools (Electric Geodesics Inc., EGI, Eugene, OR) to filter (band pass: 0.1–30 Hz), segment (300 ms epochs; 50 ms before and 250 ms after stimulus), and to detect and replace bad channels (> 200 µV). Baseline correction, artefact detection and eye blink removal were completed via independent components analysis in EEGlab (Versio14.12; Delorme & Makeig, 2004) and Matlab (Version: 2018b; The Mathworks, Inc., Natick, MA, USA; Onton, Westerfield, Townsend, & Makeig, 2006). Waveform averaging, visualization, and peak extraction for each animal per day were also conducted in EEGlab and Matlab.

### Data analysis strategy

**Fig. 2:**
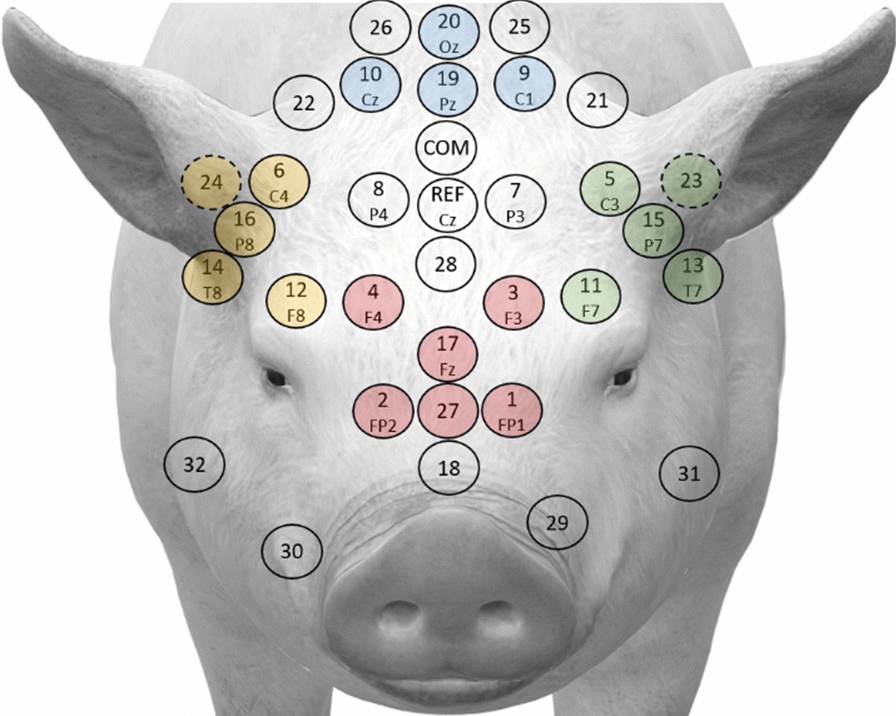
Illustration of electrode net on model piglet head with crown (blue), front (red), left (green) and right (yellow) electrode groups shown

EEG waveforms were examined to locate the first identifiable local maxima, followed by a local minima, and subsequent local maxima corresponding to the P1, N1, and P2 peaks. Prior to peak amplitude extraction of P1, N1, and P2, grand waveform averages for all three days of testing per animal were examined to determine approximate latency widows for each peak. These latencies were then used to guide peak amplitude extraction for each animal per day for electrodes representing regions of the crown (9, 10, 19, 20), front (1, 2, 3, 4, 17, 27), left (5, 11, 13, 15), and right (6, 12, 14, 16, 24) (Fig. 2). The amplitude and latency values for each group of electrodes were then averaged and used as input as a single value for statistical analysis.

### Statistical analysis

#### Part A—regular oddball clicktrain

Three-way repeated measures ANOVAs evaluated the effects of “electrode group” (4 levels: front, crown, left and right), “tone type” (2 levels: standard and target) and the repeated factor of “day” (3 levels: day 1, 2, and 3) on P1, N1, and P2, and P1-N1 and N1-P2 peak-to-peak amplitudes and latencies. Post hoc analyses involved Bonferroni tests if variances were equal based on Mauchly’s sphericity tests and Dunnett’s T3 if variances were unequal.

### Part B—alternative target pulse clicktrain

Paired t-tests for regular target pulse (1000 Hz) and alternative target pulse (250 Hz or 4000 Hz) were conducted on P1, N1, and P2, and P1-N1 and N1-P2 peak-to-peak amplitudes and latencies. All statistics were conducted using IBM SPSS Statistics Version 25 for Windows (Armonk, NY: IBM Corp.) and significance was accepted at p < 0.05.

## Results

### Part A—regular oddball clicktrain

**Fig. 3:**
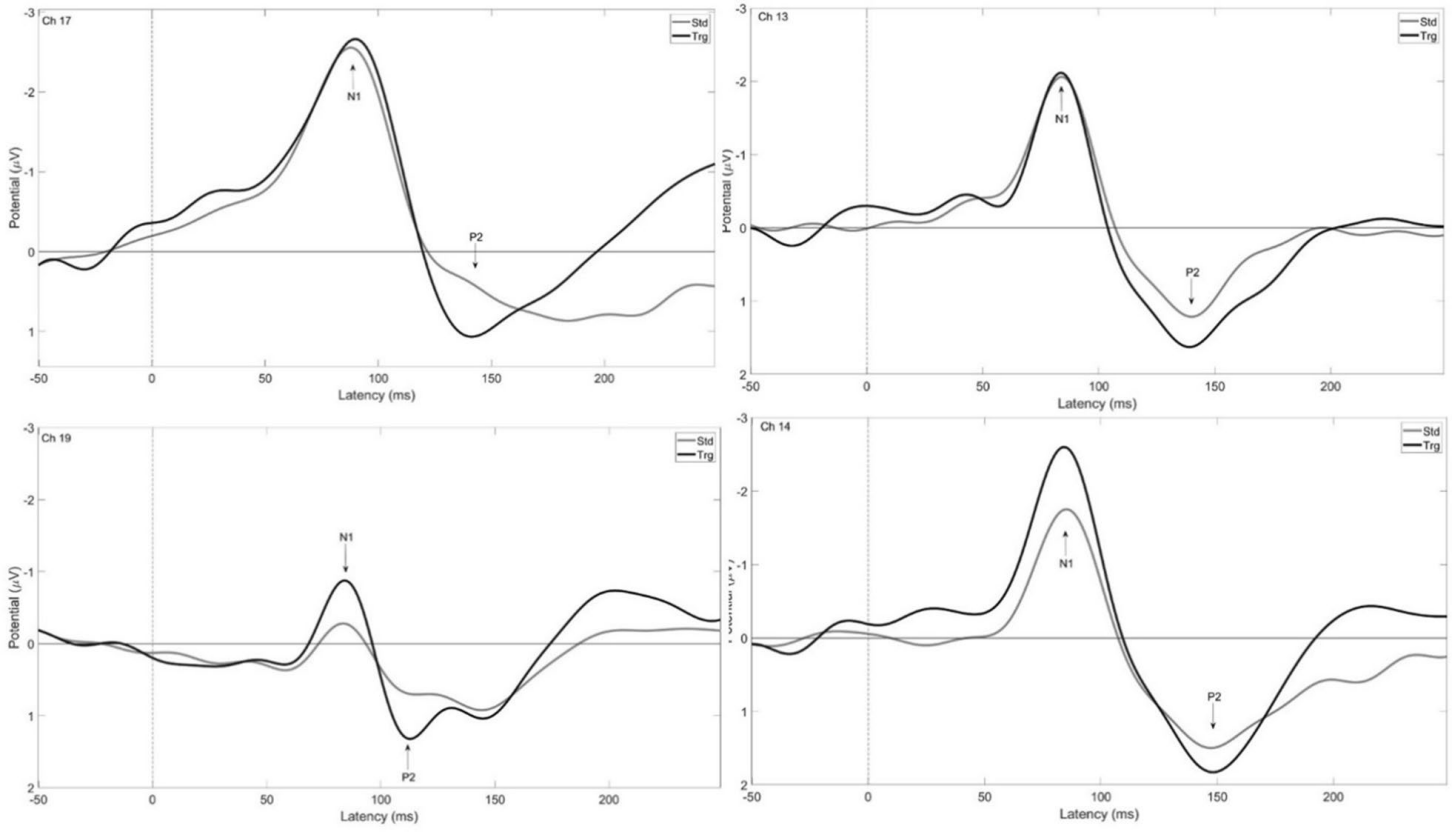
Grand averages depicting waveforms for target (black) and standard (gray) tones for single channels representing the front (Ch 17), crown (Ch 19), left (Ch 13), and right (Ch 14) for regular auditory oddball tests

Grand averages for target and standard waveforms are presented in Fig. 3. A single electrode is shown to represent the different electrode groups: 17 for front, 19 for crown, 13 and 14 for left and right, respectively. The N1 and P2 amplitudes had consistent negative and positive values however; distinguishable P1 local maxima were not always present or did not have a positive value across electrodes, animals, and test days (Fig. 3). Therefore, P1 values were not used in the statistical analysis and the dependent variables were limited to N1, P2, and N1-P2 peak-to-peak amplitudes and latencies.

**Fig. 4:**
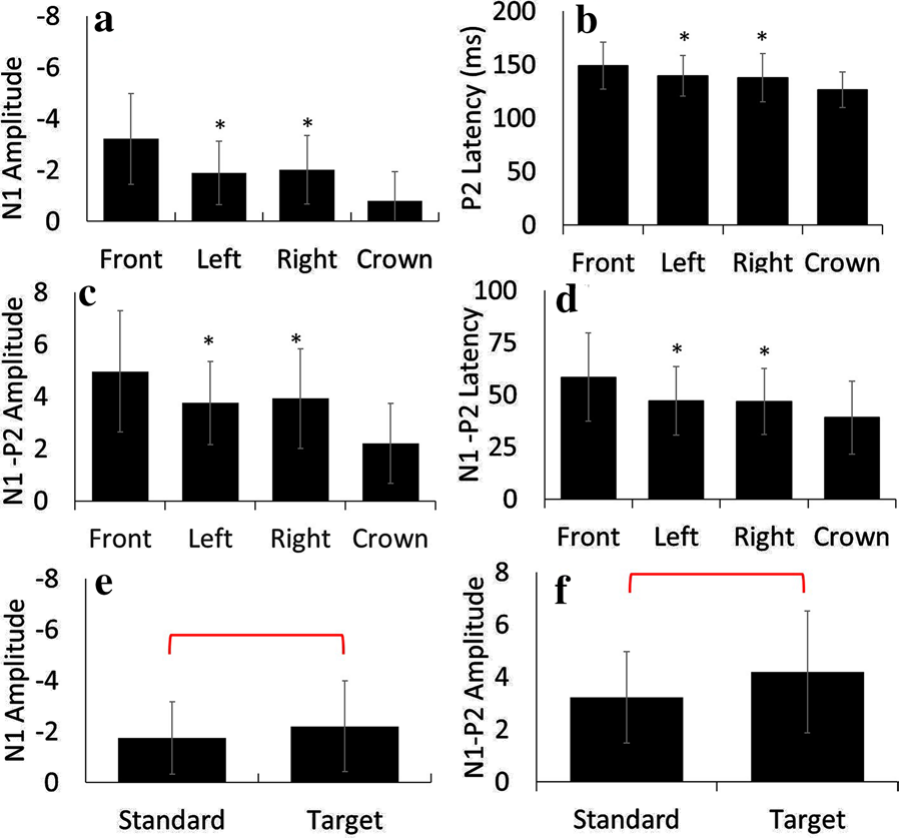
Significant findings for electrode group with N1 amplitude (**a**), P2 latency (**b**), and N1-P2 amplitude (**c**) and latency (**d**). All comparisons were statistically significant except where ‘*’ denotes statistically similar results. Significant findings for tone type are illustrated for N1 (**e**) and N1-P2 (**f**) amplitudes with significant differences are illustrated with an overlaying bar

A summary of amplitudes and latencies for healthy 4 week-old piglets across all three days tested is presented in Table 1 as means and standard deviations for each electrode group. There were significant main effects for tone type (F(1,11) = 5.4, p=0.041, ηp^2^ = 0.328) and electrode group (F(1.8, 20.1) = 30.7, p < 0.01, ηp^2^ = 0.736) for N1 amplitudes and N1-P2 amplitudes (electrode group: (F(3,33) = 22.9, p < 0.01, ηp^2^ = 0.675; tone type: (F(1,11) = 18.3, p = 0.001, ηp^2^ = 0.624). Post hoc analyses revealed similar effects for both dependent variables, where the crown had the lowest values, followed by the left and right regions, which were not significantly different from each other, and the front electrodes had the largest values (p < 0.05). For tone type, target tones produced greater amplitudes than standard tones (p < 0.05). Similarly for peak latencies, there was a significant main effect of electrode group (F(3,33) = 14.7, p < 0.01, ηp^2^ = 0.571) on P2 latency and N1-P2 latency (F(3,33) = 16.7, p < 0.01, ηp^2^ = 0.603). Post hoc analyses revealed that the crown had the shortest latencies, followed by left and right regions, and front with the longest latencies (p < 0.05). A depiction of significant results are shown in Fig. 4.

**Table 1:** Summary table of means and standard deviations of N1 and P2 amplitudes (µV) and latencies (ms) for the target and standard tones across electrode groups

	Electrode group	Amplitude (µV)		Latency (ms)	
		Target	Standard	Target	Standard
N1	Front	− 3.5 (2.0)	− 2.9 (1.5)	92.1 (11.8)	89.2 (11.2)
	Crown	− 1.0 (1.3)	− 0.6 (0.9)	87.0 (13.1)	88.0 (15.7)
	Left	− 2.0 (1.3)	− 1.8 (1.2)	93.5 (12.9)	91.6 (13.4)
	Right	− 2.3 (1.5)	− 1.7 (1.1)	91.7 (17.4)	90.3 (16.4)
P2	Front	2.0 (2.2)	1.5 (1.3)	147.9 (18.3)	150.5 (25.5)
	Crown	1.7 (1.4)	1.1 (1.1)	124.4 (13.3)	128.9 (19.6)
	Left	2.1 (1.8)	1.6 (1.3)	140.1 (17.7)	139.3 (20.3)
	Right	2.1 (1.7)	1.7 (1.2)	138.3 (20.8)	137.4 (24.2)

### Part B—alternative target pulse clicktrain

**Fig. 5:**
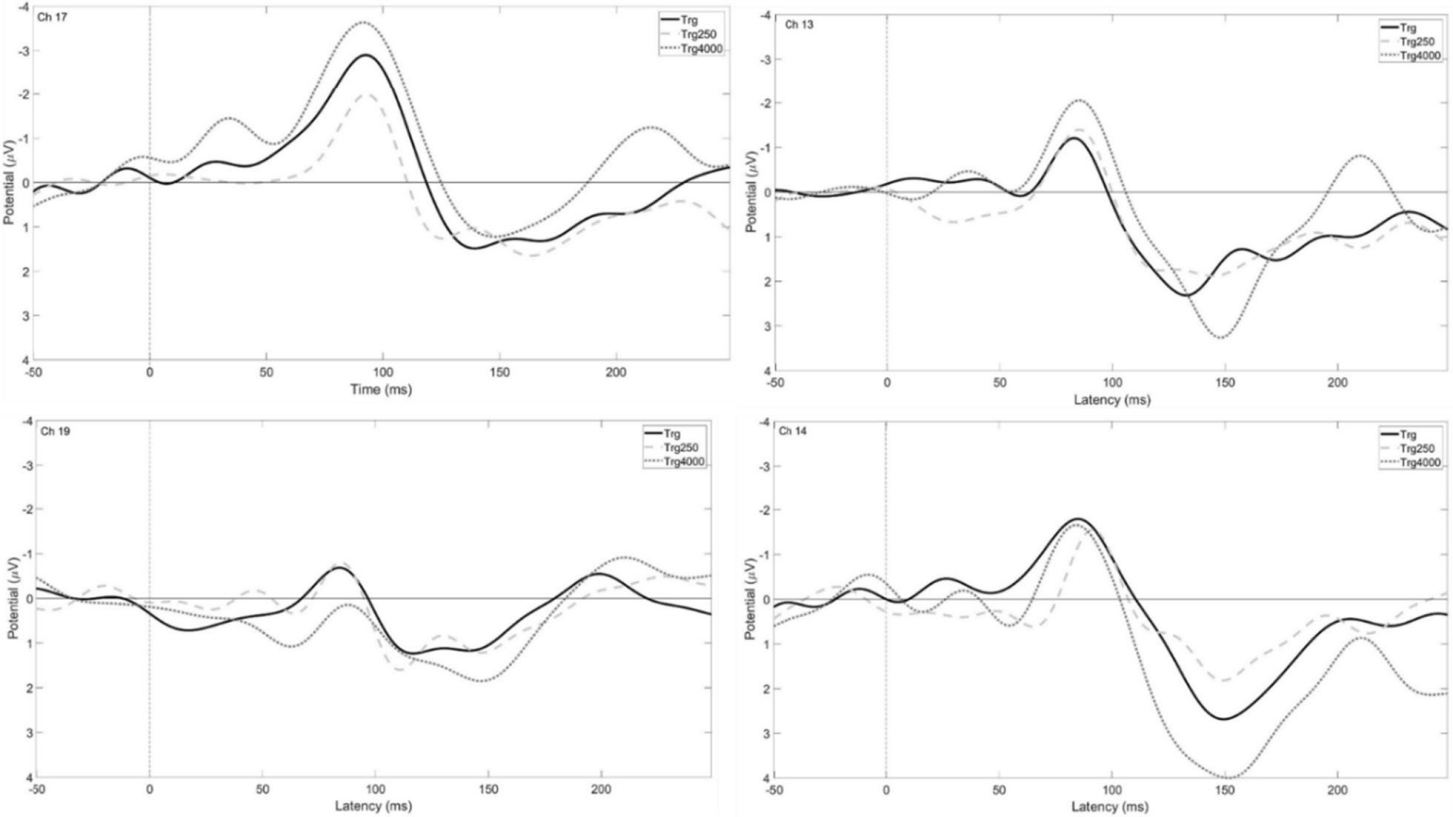
Grand average waveforms for Low (250 Hz), Regular (1000 Hz) and High (4000 Hz) target tones

**Fig. 6:**
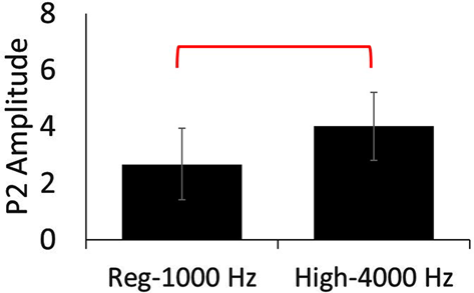
P2 amplitude results for regular 1000 Hz target tone and high 4000 Hz target tone for the front electrode group. Significance is indicated with an overlaying bar

Grand averages for low, regular, and high target waveforms are presented in Fig. 5. A single electrode is shown to represent the different electrode groups: 17 for front, 19 for crown, 13 and 14 for left and right, respectively. A summary of amplitudes and latencies for low and high target tones are presented in Table 2 as means and standard deviations for each electrode group. Based on the findings from *Part A*, the statistical analyses were limited to N1, P2, and N1-P2 peak-to-peak amplitudes and latencies for target tones. Low target tones (250 Hz) did not produce significantly different results than target tones in the regular tests (1000 Hz), however high target tones (4000 Hz) were found to produce significantly greater P2 amplitudes (Fig. 6) than regular target tones (1000 Hz) for the right electrode group (*t*[4] = -4.03, *p* = 0.016).

**Table 2:** Summary table of means and standard deviations of N1 and P2 amplitudes (µV) and latencies (ms) for the low and high target tones across electrode groups

	Electrode group	Amplitude (µV)		Latency (ms)	
		Low (250 Hz)	High (4000 Hz)	Low (250 Hz)	High (4000 Hz)
N1	Front	− 2.8 (1.7)	− 3.3 (1.4)	96.2 (4.9)	90.4 (7.3)
	Crown	− 0.7 (1.7)	− 0.1 (1.1)	92.8 (16.3)	87.4 (3.5)
	Left	− 1.7 (1.4)	− 1.8 (1.4)	92.8 (23.0)	93.9 (9.0)
	Right	− 1.8 (1.2)	− 1.2 (0.7)	98.7 (7.4)	89.7 (11.8)
P2	Front	1.4 (0.6)	3.2 (1.3)	155.6 (10.6)	157.1 (30.2)
	Crown	1.7 (1.2)	2.2 (1.1)	132.4 (23.2)	135.3 (15.3)
	Left	1.8 (1.1)	3.4 (1.4)	151.0 (24.2)	156.9 (26.6)
	Right	2.0 (0.8)	4.0 (1.2)	155.3 (12.9)	146.8 (13.0)

## Discussion

A two-tone auditory oddball paradigm was used to characterize ERPs in healthy 4-week old piglets. N1 and P2 local minima and maxima were the two features of the electrical potential waveform that had consistent negative and positive values occurring at N90 (82–92 ms) and P140 (124–150 ms), respectively. It is possible that these waveform responses are congruent to the N130 at 132 ms and the P160 at 156 ms reported for a two-tone auditory oddball paradigm studied in older (12–14 months) male Gottigen minipigs [37]. In the 4-week old piglets studied here, infrequent target stimuli elicited greater waveform responses than frequent standard tones. This is in accordance with findings reported by Arnfred, Lind [37] for older male minipigs, and also in children 4–13 years old [16, 38] and adults [18] for passive listening to an auditory oddball paradigm.

Regional differences were observed for N1 and P2 responses as noted by the averaged values for each electrode group. Electrodes capturing activity at the front had the greatest waveform responses, followed by the right and left regions, with the crown region demonstrating the smallest waveforms. These findings are consistent with those found in humans, where the fronto-central region reflected the greatest activity for target tones in an auditory oddball paradigm [13, 14, 18]. Similarly, this finding was also consistent in older (12–14 month) male Gottingen minipigs where frontal channels demonstrated greater activity than posterior channels [39]. Activity observed in the left and right electrode groups in this study may be reflective of involvement of the early central auditory pathways and auditory cortex in response to the identification and perception of sound [12]. The left and right electrode groups are regions close to the ears and auditory cortex where auditory regions have been reported to be located near the lateral fissure in miniature swine [40]. Reduced activity at the crown is likely due to the electrodes covering this region are located behind the ears and toward the back of the neck. It is unlikely that cortical activity related to auditory processing are detected in this area (Fig. 2).

Regular auditory oddball tests were collected for three non-consecutive days for each animal in this cohort. There were no significant main effects of test day; therefore, two-tone auditory oddball tests elicit consistent day-to-day responses and these methods area repeatable in 4-week old piglets. Furthermore, results from the alternative target tone tests showed that modifying the pitch of the target tone was not found to influence the N1 amplitudes; however, the higher 4000 Hz target tones produced greater P2 amplitudes than the 1000 Hz target tones. Like the N1, the P2 has also been associated with early attention allocation and is also considered a reliable indicator of auditory stimulus processing in humans [12].

A direct limitation of this study are that ERP data were extracted from a subset of 20 electrodes from the total 32 that were concurrently used to collect EEG. The method of peak amplitude and latency extraction corresponding to N1 and P2 features of the middle latency response are common to the greater auditory processing literature, however, due to the complexity of neural processes and cognition, these results may only capture a single aspect of multifaceted auditory processing. In addition, age specific responses have been noted in the human literature where the amplitude and latency responses are a function of age and brain maturation, where responses for adults are unique from infants, children, and adolescent age groups [41–43]. It is likely that auditory responses in piglets are similarly affected by age and maturational processes, therefore, these findings are specific to the 4-week old piglet. The 4-week old piglet is a common animal model used to represent traumatic brain injury in children due to parallels in brain composition during early development [30, 33, 44].

## Conclusions

An auditory oddball paradigm elicited consistent N1 and P2 middle latency responses in healthy 4-week old piglets. Infrequent target tones elicited greater N1 and N1-P2 amplitudes than standard tones, which is in alignment with other pig studies and the human literature for children and adults. Greater activity toward the front electrode group in 4-week old piglets is consistent with maximal fronto-central activity associated with auditory deviant sound processing in humans. There were no effects of test day; therefore, two-tone auditory oddball tests elicit consistent day-to-day responses. This clinical assessment was successful in the immature large animal model.

## Data Availability

The datasets used and/or analysed during the current study are available from the corresponding author on reasonable request.

## Abbreviations

AAALAC: Association for Assessment and Accreditation of Laboratory Animal Care
EEG: Electroencephalography
ERP: Event-Related Potential
GSN: Geodesic Sensor Net
IACUC: Institutional Animal Care and Use Committee
mTBI: Mild Traumatic Brain Injury
TBI: Traumatic Brain Injury
